# Principles of dose-setting in toxicology studies: the importance of kinetics for ensuring human safety

**DOI:** 10.1007/s00204-021-03155-4

**Published:** 2021-10-08

**Authors:** C. J. Borgert, C. Fuentes, L. D. Burgoon

**Affiliations:** 1 Applied Pharmacology and Toxicology, Inc., Gainesville, FL USA; 2 Center for Environmental and Human Toxicology (CEHT), Department of Physiological Sciences, University of Florida College of Veterinary Medicine, Gainesville, FL USA; 3 Department of Statistics, Oregon State University, Corvallis, OR USA; 4 Raptor Pharm and Tox, Ltd., Apex, NC USA

**Keywords:** Kinetic Maximum Dose (KMD), Maximum Tolerated Dose (MTD), Pharmacokinetics, Toxicokinetics, Toxicology study design

## Abstract

Regulatory toxicology seeks to ensure that exposures to chemicals encountered in the environment, in the workplace, or in products pose no significant hazards and produce no harm to humans or other organisms, i.e., that chemicals are used safely. The most practical and direct means of ensuring that hazards and harms are avoided is to identify the doses and conditions under which chemical toxicity does not occur so that chemical concentrations and exposures can be appropriately limited. Modern advancements in pharmacology and toxicology have revealed that the rates and mechanisms by which organisms absorb, distribute, metabolize and eliminate chemicals—i.e., the field of kinetics—often determine the doses and conditions under which hazard, and harm, are absent, i.e., the safe dose range. Since kinetics, like chemical hazard and toxicity, are extensive properties that depend on the amount of the chemical encountered, it is possible to identify the maximum dose under which organisms can efficiently metabolize and eliminate the chemicals to which they are exposed, a dose that has been referred to as the kinetic maximum dose, or KMD. This review explains the rationale that compels regulatory toxicology to embrace the advancements made possible by kinetics, why understanding the kinetic relationship between the blood level produced and the administered dose of a chemical is essential for identifying the safe dose range, and why dose-setting in regulatory toxicology studies should be informed by estimates of the KMD rather than rely on the flawed concept of maximum-tolerated toxic dose, or MTD.

## Introduction

This article explains why it is important for regulatory toxicity testing strategies to incorporate pharmacokinetics and toxicokinetics (hereafter, PK/TK), which many consider to be one of the most important scientific developments in pharmacology and toxicology of the last century. PK/TK encompasses the measurement and elucidation of mechanisms by which organisms interact with chemicals in their environment, i.e., the way organisms absorb, distribute, metabolize (transform), and eliminate chemicals from the body, often referred to as “ADME.” This field of inquiry has advanced our understanding of both the adverse and therapeutic effects of drugs and chemicals on living organisms (Dunnington et al. 2018; Webborn 2014). PK had its origins in the mid-twentieth century (Wagner 1981) and as the field matured, grew, and became well accepted, pharmacokinetic understanding led to numerous medical advancements. To list just a few, these include understanding the kinetic determinants of drug sensitivity and resistance (McCallum and Sloan 2017), the development of sophisticated methods of drug delivery that ensure effective concentrations of medication at the therapeutic target organ or tissue while reducing the administered dose required for efficacy (Glassman and Muzykantov 2019), the development of pharmacogenomics (Nakajima and Yokoi 2005) and individualized pharmacotherapy (Magliocco et al. 2020), and the possibility of reducing drug development costs through pharmacokinetic modeling and simulation (Feng and Leary 2017). Although beyond our scope to elaborate further, it would be difficult to overstate the importance of pharmacokinetics to modern pharmacotherapy.

Similarly, TK has enabled many advancements that have been instrumental in toxicology beyond the obvious importance of clarifying the rates at which chemicals are absorbed and eliminated (Andersen 1981). Toxicokinetics has enabled the quantification of chemical bioavailability by different routes of exposure and helps to clarify the modes of action (MoAs) by which route-dependent toxicity occurs. Both can be critically informative for defining safe levels of exposure. The use of physiologically based pharmacokinetic (PBPK) models to conduct tissue dosimetry-based risk assessments was first described for methylene chloride (Andersen et al. 1987), and was recently updated with carboxyhemoglobin and genomic modules (Andersen et al. 2017). These modules were significant for using PBPK modeling to link carbon monoxide formation to the dose–response for genomic effects, thereby reframing the likely mode of action for methylene chloride animal tumorigenicity as high-dose-specific and kinetically irrelevant for human cancer risks at feasible human exposures. For decades prior to this, high-dose specific methylene chloride rodent tumorigenicity was attributed to a MoA involving formation of genotoxic reactive metabolite(s) derived from a methylene chloride glutathione conjugate metabolite. However, the Andersen et al. paper used both PBPK modeling and genomic data to challenge that MoA paradigm, and shift it to a more toxicologically plausible association with carbon monoxide, the primary oxidative metabolite of methylene chloride. The advancements made with methylene chloride were soon followed by many other PBPK-based improvements in risk assessments (summarized in Andersen et al. 2021), which collectively established the importance of PBPK modeling for interpreting the relevance of high-dose-specific animal toxicity to human risk. This work further underscored the fact that adverse effects observed only at animal blood/tissue concentrations that are substantially different from human blood/tissue concentrations resulting from realistic human exposure scenarios provide little useful information for understanding human toxicity and risk.

One of the more prominent regulatory uses of kinetic-based toxicology is the regulation of chloroform; chloroform produces liver and kidney tumors in rodents, but these tumors arise by a MoA that is dependent on achieving daily peak plasma concentrations sufficient to produce cytotoxicity in those organs. Bolus administration of high doses of chloroform by gavage satisfies the kinetic requirement for cytotoxic peak plasma concentrations whereas even higher daily doses administered in drinking water do not. This kinetic difference is critical and informs which type of study design, and consequently, which chronic effects of chloroform in rodent toxicology studies might be relevant for hazard identification and human risk assessment (reviewed in Borgert et al. 2015).

Other major contributions of TK involve examination of the processes that determine tissue doses and tissue interactions of chemicals. Some notable examples include TCDD accumulation in liver and induction of CYP1A2 as a binding protein (Andersen 1981); identification of the key role of kidney resorption of perfluoro-octanoic acid (PFOA) and its persistence in the body (Andersen et al. 2006); distinguishing endogenous formaldehyde, its levels in various tissues and its production by various biochemical pathways from exogenously inhaled formaldehyde and its lack of systemic absorption (Andersen et al. 2010; Campbell et al. 2020); differentiating contributions to tissue manganese concentrations from natural background versus occupational or community exposures (Schroeter et al. 2011, 2012; Taylor et al. 2012), and; assessment of route-of-exposure-dependent differences in chemical distribution and kinetics (Campbell et al. 2017; McMullin et al. 2016; Slikker et al. 2004a, b). PBPK models have improved the understanding of mixture toxicology (Dennison et al. 2003; Haddad et al. 2001; Yang et al. 2004), enabled life-stage-specific modeling of chemical exposures (Clewell et al. 2004; Loccisano et al. 2013; Yoon et al. 2011), and provide the foundation for reverse-dosimetry to understand the relevance of biomonitoring data and biomarkers in various biological media (Clewell et al. 2008). TK and PBPK modeling enables in vitro to in vivo extrapolation (IVIVE) to establish the human risk relevance of chemical concentrations that produce responses in high throughput and in vitro test systems compared with blood/tissue concentrations resulting from reasonably foreseeable human exposures (Thomas et al. 2013). Most significantly for 21st  Century Toxicology, TK enables the development of biokinetic methods to predict in vivo effects from in vitro data and to improve the basis for in vitro to in vivo dose extrapolations (Blaauboer 2010; Groothuis et al. 2015). Finally, any such list would be incomplete without mentioning that kinetic understanding can uncover an oft-overlooked source of bias in epidemiological studies that attempt to link health outcomes to putative biomarkers of disease and toxicity without considering or controlling for the potential confounding of biomarker measurements that may arise from disease-induced TK alterations (Andersen et al. 2021).

The above is but a brief and incomplete discussion of the important advancements in pharmacology and toxicology made possible by rigorous application of PK/TK, but it is included to expose the naiveté of suggesting that PK/TK data are insufficient or methodologically inferior to descriptive toxicology for selecting doses in toxicological studies. Although some regulatory guidance documents on dose setting acknowledge the potential importance of kinetics, there remains considerable resistance to the advancements that can be realized through use of PK/TK. As explained further in this review, such resistance would appear to derive from adherence to overly restrictive definitions and narrowly constrained interpretations of the salient issues (e.g., that a hazard identified at high doses is relevant for all doses and can be used to ensure safety) rather than from any legitimate argument as to why proper application of PK/TK is not a rational approach to dose-setting for toxicological investigations. These factors may also underly reluctance to depart from the traditional, standardized approach to dose-setting in regulatory toxicology studies that relies on the concept of a maximum-tolerated dose.

Since the 1970’s, dose selection for regulatory toxicology studies has relied on the demonstrably flawed concept of “maximum-tolerated dose,” usually denoted “MTD” (Borgert et al. 2015; Freedman and Zeisel 1988; Gaylor 2005). Briefly, acute or short-term toxicity testing is used to define dose-levels that produce overt toxicity, and those dose levels are then reduced by the least amount necessary to allow animals to survive through the course of longer-term toxicity tests. Typically, at least one dose administered to animals for the duration of sub-chronic, multi-generational, and life-time toxicity tests are required to produce either observable but survivable overt toxicity or no more than a ten percent reduction in body weight gain. Such doses are considered to be “tolerated” by the test species—thus, the “MTD” designation—despite the fact that impaired health may well occur secondary to these so-called “tolerated” doses by mechanisms such as nutritional deficiencies, stress, delayed development, and endocrine abnormalities associated with reduced body weight gain (Gaylor 2005; Marty et al. 2018).

The rationale for dosing at the MTD is to increase the statistical power of a study for detecting low-incidence effects, which would otherwise require a drastic increase in group sizes. However, the supposed power advantage of MTD-observed toxicity does not and cannot compensate for the inability of small group sizes in toxicity tests to predict whether adverse responses might occur at, often, very much lower doses produced by typical human exposure levels. The incongruity of that reasoning seems self-evident, but to explain briefly, if group size and dose level were statistically interchangeable, one could test the expected incidence of water toxicity among one million people who consume 5 L daily for a lifetime by administering 50 L of water to 100 people every day for a year. Clearly, one cannot assume a linear relationship between biological responses and dose over the entire range of doses that can be tested, up to the MTD, and that responses observed only at the MTD are nonetheless representative of hazard at all, even much lower, exposure levels. Decades of toxicology testing and TK evaluation have shown that this assumption is incorrect for many chemicals (Slikker et al. 2004a, b).

To understand why TK is critical for rational dose-setting and interpretation of regulatory toxicity testing, it is important to appreciate that an explicit assumption underlying this publication is that the role of mammalian toxicology in chemical safety assessment is to characterize the conditions under which chemicals can be used safely, i.e., those conditions devoid of relevant hazards, which thereby pose negligible risks of adverse effects on human health, and to define the limits of those conditions so that relevant hazards and adverse consequences can be avoided. The obvious exception to this goal is that acute toxicity testing at and above the MTD may be necessary to provide information to treating physicians who must understand the potential clinical presentation and target organs affected by acute poisoning events. Otherwise, although discovering all possible hazards and adverse effects of a chemical under all testable conditions may be of scientific interest in other realms of toxicology, repeat-dose toxicity studies at the MTD have no practical utility in drug and chemical safety assessment or in the regulatory context. As explained herein, the accuracy and integrity of safety assessments are often undermined by the attempt to characterize all adverse effects of a drug or chemical irrespective of whether the administered doses are quantitatively or kinetically relevant to actual exposures.

## Principles and concepts

To achieve the regulatory goal of ensuring that chemical uses are limited to the conditions under which exposures are safe, dose-setting for regulatory toxicology studies should be aimed at identifying and characterizing the dose range at which adverse effects are unobservable by validated test methods. To achieve this efficiently, we would propose that the administered doses should cover the range from very low (e.g., the low end of the estimated human exposure level) up to, but not exceeding, the dose that produces either:

- Adverse effects and irreversible changes that must be assumed to be adverse.
- A dose-disproportionate alteration in the relationship between the administered dose and the blood level of the chemical.

We acknowledge that our proposal challenges the status quo of current regulatory practice and may meet resistance because of that fact alone. Some may object to testing doses as low as we propose, finding it preferable to begin toxicity testing at doses 10–100 times above the estimated human exposure level to increase the chances of identifying a NOAEL and to avoid the excessive conservatism that can ensue when a NOAEL is not defined. As discussed herein, testing human-relevant doses on the low end is important to ensure that significant kinetic changes are identifiable. An alternative approach to identification of a NOAEL will be addressed in a subsequent paper, but this paper focuses on selection of the top dose for regulatory toxicity studies. Some may also object to testing doses no higher than those that alter kinetics; however, it is important to recognize that our proposal does not differ from standard regulatory dose-setting for chemicals that exhibit uniform kinetics from low to high doses. The remainder of this paper explains the rationale for our recommendations using examples from well-characterized drugs.

### Why identify and characterize the no-effect dosage range?

#### Practicality

It is often assumed that the purpose of guideline toxicology studies is to identify all possible adverse effects and to characterize their dose–response relationships, but we would contend that in fact, current toxicology study designs are a compromise that attempt to identify the safe dose range as well as to characterize adverse effects that are within, typically, 100–1000-fold greater than expected human exposures, a dual focus that limits the ability of toxicology studies to serve either purpose well. In practice, MTD doses may exceed human doses by even greater magnitudes, further eroding plausible relationships to foreseeable human exposures. If comprehensive testing for adverse effects were to be done thoroughly, each type of toxicology study would need to incorporate many different treatment arms tailored to examine all organ systems and processes within the dose ranges that the chemical affects each system. For example, a reproductive toxicology study that attempts to test for effects on both anogenital distance and fertility in the offspring would need to employ much larger animal numbers and more treatment groups than currently required because statistical optimization would be different for detecting biologically relevant changes in these different endpoints. Adequate dose–response characterization would then require distinct administration protocols and separate control groups for each adverse effect tested in that type of study, as well as many more dose levels than currently required by OECD, U.S. EPA, and other international regulatory test guidelines. This would expand the use of animals unnecessarily, raise the complexity of many types of toxicology studies, and hence, increase costs and the potential for human error.

Focusing toxicology studies exclusively on the safe dose range rather than on the dose range that produces toxicity would be a superior approach for several reasons. Above all, it is practical. Human exposures to chemicals are not intended to pose hazards or produce adverse effects; to the contrary, when exposure to chemicals occurs, it is intended to be non-hazardous and without adverse effects. Therefore, it is logical that the highest priority of toxicity testing should be to identify and characterize the doses and conditions that meet this intent. Focusing on the safe dose range is also necessary from a logistical standpoint because ensuring safety requires that the various biological targets that could be adversely affected by a chemical are, in fact, not affected under foreseeable conditions of exposure.

Assuring that the dose range and conditions have been identified under which a chemical does not affect even one of its many possible biological targets is a fundamentally different objective, and arguably a more difficult challenge, than merely identifying that an adverse effect can be observed at some dose, irrespective of its relevance to actual conditions of use and foreseeable exposures. In fact, it is axiomatic and assured that all chemicals will produce an adverse effect at some dose because all chemicals are toxic (i.e., hazardous) under some conditions. Since the assurance of *no adverse effects* is the most critical goal of toxicology testing, it is prudent to expend sufficient resources to ensure that those conditions are thoroughly defined rather than attempting to also address questions less relevant to safety, such as characterizing the various effects that might occur at doses beyond the safe dose range.

Even when more resources are expended than are typically available for chemical risk assessment, it can be very difficult to dismiss the potential human relevance of effects observed experimentally in high-dose animal toxicology studies without information about the TK relevance of those doses. Formaldehyde and chloroform are prominent cases of this problem that still engender controversy and debate. If the doses selected for key studies on these chemicals had been initially informed by their TK behavior, human cancer hazards would not have been inferred because the tumors produced by these chemicals in animals can occur only with repeated exposure to cytotoxic concentrations, conditions not foreseeable under any human circumstance. The regulatory history of these two chemicals clearly attests to the increased efficiency and certainty that can be provided by consideration of TK in determining the doses appropriate for regulatory toxicology studies.

#### Achievability

Notwithstanding philosophical arguments against absolute proof of a negative proposition, defining a no-effect dose range is achievable. When toxicology studies are properly designed, statistical approaches can be used to determine how confident one can be that the adverse effect will not occur within a particular dose range. Properly designed studies should include a consideration of dose-dependent TK, as statistical approaches applied to analysis of dose–response curves that include doses saturating TK will not provide for an estimate of confidence that adverse effects are absent at realistic or reasonably foreseeable human exposure levels.

#### Fit for purpose

It is also important to appreciate that different goals drive the design of different types of toxicology studies and for this reason, the administered doses and the endpoints measured often vary considerably between acute, sub-chronic, and chronic toxicology studies. Acute tests are intended to identify immediate effects indicative of overt poisoning and do not assume that a steady-state blood level has been achieved. Since blood levels are considered a surrogate for, and directly affect the target tissue concentration, which is the critical determinant of toxicity, assumptions about steady state are important. Sub-chronic studies are aimed at identifying adverse effects of repeated dosing, and chronic tests are intended to allow identification of subtle types of adverse effects that require long periods of time to develop or that relate to growth or development that occurs over specific periods of the lifespan. Both sub-chronic and chronic toxicology studies, but not acute studies, assume that steady-state blood levels, or at least a consistent daily fluctuation of the chemical concentration in blood, has been achieved. Although repeat-dose studies for most chemicals, except those that bioaccumulate extensively or have long half-lives, may indeed achieve steady state, that assumption is usually not verified in toxicology studies because the dosing regimen is based on the MTD rather than on pharmacokinetic information. Verifying the underlying assumptions of a test is an important aspect of data quality for any scientific study and is one critical advantage of using pharmacokinetic/toxicokinetic information to establish dose-setting.

Arguments have been made that a variety of scenarios may result in human exposures higher than currently occur, and, therefore, that dose selection for toxicology studies based on the KMD, and even the MTD, would be too low to identify relevant human health effects. Those scenarios are said to include future chemical uses that produce human exposures higher than currently occur, as well as the possibility of personal protective equipment failure in the workplace, accidental chemicals releases, and intentional or unintentional overexposures from product misuse (Heringa et al. 2020a; Woutersen et al. 2020). This argument is not compelling for several reasons.

Given that human poisoning events and accidental chemical spills are highly unlikely to occur on a repeated daily basis over extended timeframes such as those tested in sub-chronic and chronic toxicity studies, dosing at or above the MTD makes no sense other than for acute toxicity studies. Acute toxicology studies provide information useful for understanding chemical handling precautions by chemical workers and treatment of acute poisoning and accidental exposure, but as will be explained in subsequent sections, attempting to glean such information using excessively high doses in sub-chronic and chronic studies is not only ineffective but can also compromise the information intended to be supplied by those studies. Furthermore, dose selection for repeat-dose studies based on the MTD, and even on the KMD (which might reduce the top dose by a factor of 10 from the MTD), generally results in the highest tested dose exceeding realistic human exposures by orders of magnitude based on chemicals whose human exposure is well characterized.

Moreover, regulatory exposure limits are established by application of uncertainty factors of at least 10 ×, and usually 100 ×–1000 × or higher, to lowest-observable and no-observable adverse effect doses identified from studies that measure multiple endpoints across different life stages (e.g., reproduction, development, life-time exposure). Thus, the likelihood seems remote that MTD-based dose selection would miss adverse effects that are relevant to human health. Finally, if rather than attempting to identify all adverse effects of a chemical irrespective of dose, toxicity testing focused on identifying the hazard-free/toxicity-free dose range more unequivocally and translating those doses to humans by use of toxicokinetics, as we propose here, arguments that favor higher-than-MTD dosing would be rendered even more tenuous.

### Why test doses up to the point of either toxicity or altered toxicokinetics, but not beyond?

Defining the safe dose range may be biologically incongruent with characterizing the various adverse effects that occur across the entirety of the toxic dose range. This derives from the fact that toxicity is a dynamic process with many causal factors, one of the most critical being toxicokinetics. Neither toxicity nor its regulatory antecedent “hazard” arise purely from the constituent atoms or the molecular structure of a chemical, and each molecule of the chemical does not exhibit toxicity or hazard irrespective of the number of molecules present. Thus, toxicity and hazard are not inherent or intrinsic to the chemical. In the vernacular of chemistry, toxicity is not an “intensive” property of a chemical. To the contrary, toxicity and hazard are “extensive” properties, meaning that in addition to a chemical’s structure and physical nature, toxicity and hazard depend upon the quantity of the chemical encountered, the route of exposure and the conditions under which a chemical is encountered, and other factors such as the species, age, sex, behavior, and other characteristics of the organism exposed to the chemical (McCarty et al. 2020). Thus, the dose range and conditions under which a chemical produces toxicity may provide little useful information about the dose range and conditions necessary to assure a lack of relevant hazard or adverse effects.

In fact, because both overt toxicity and the MoAs underlying it are dose-dependent (Slikker et al. 2004a, b), focusing on the toxic dose range lacks dosimetric quantitative relevance to realistic and foreseeable human exposures and thus, provides no information necessary for assuring safety. In some instances, the mechanisms induced as a result of high-dose testing may actually obscure the mechanisms and endpoints that are most important for assuring safety because the latter can be affected indirectly and artefactually by the former (e.g., Marty et al. 2018). These facts are not controversial and occur with most chemicals. Consider, for example, one of the oldest and most widely used pharmaceuticals in history, acetyl salicylate, known commonly as Aspirin^®^ (Cadavid 2017).^1^ Like all chemicals, the effects of aspirin and the MoAs by which its effects are produced in humans depends upon many factors, but three of the most important are the route of administration, the administered dose, and the blood level.

### Example #1: aspirin

Aspirin has exceedingly low bioavailability by the dermal route and biologically meaningful systemic effects do not occur by this route of administration without use of a vehicle such as dimethylsulfoxide (DMSO). At relatively low blood concentrations that can be achieved by oral administration of 30–100 mg daily, aspirin exhibits potent anti-thrombotic activity through acetylation of various enzymes important in platelet aggregation, primarily cyclooxygenase 1, which is essential for synthesis of thromboxane A2 (Cadavid 2017; Undas et al. 2007). Through this MoA, low-dose aspirin produces anti-thrombotic and anticoagulant effects, which contributes cardioprotection in patients at risk for cardiovascular events. At slightly higher doses, aspirin is therapeutically effective as an analgesic and anti-inflammatory agent through a variety of mechanisms primarily involving reduced prostacyclin and prostaglandin synthesis via cyclooxygenase-2, an enzyme for which aspirin also has affinity and exhibits inhibitory activity. This mode of action also accounts for gastrointestinal irritation and bleeding, the most common side effect of aspirin at therapeutic doses (Lanas and Scheiman 2007).

At therapeutic dosages, the liver metabolizes salicylates to inactive products through processes that occur by approximately first-order (Michaelis–Menten) kinetics. The inactive metabolites are then excreted via the kidney in urine, with overall elimination kinetics also approximating a first-order process. At therapeutic doses, aspirin changes acid/base balance and electrolytes resulting in a respiratory alkalosis that is compensated via normal renal and respiratory functions (Clinical Pharmacology 2021). Plasma half-lives of salicylate are 2–12 h at low to high therapeutic doses, but at supratherapeutic doses, these pathways become saturated, changing the kinetics of elimination from simple first-order to zero order, which leads to the accumulation of salicylate levels in the blood. As blood salicylate rises well above the therapeutic range of up to 30 mg/dL (Pearlman and Gambhir 2009), a high anion-gap metabolic acidosis develops that affects a number of critical organ systems and can be lethal (Abramson 2020; Pearlman and Gambhir 2009). According to Pearlman and Gambhir (2009): “*The saturation of the enzymes of elimination of salicylate is an important component in the development of chronic salicylate toxicity and is responsible for the increased serum half-life and prolonged toxicity.*

Differences between the therapeutic versus higher-dose toxic MoAs for aspirin illustrates several points that underscore our proposed principles of dose-setting. Clearly, high anion-gap metabolic acidosis is not an intrinsic or inherent property of aspirin because it is not observed to any degree at therapeutic blood levels, yet it is indeed the most life-threatening of its potential adverse effects and the one observed most consistently at high doses. Second, salicylate doses that saturate the capacity of enzymes to metabolize and eliminate it by first-order Michaelis–Menten kinetics introduce biochemical and physiological conditions that lead to dose-disproportionately higher salicylate blood levels. Third, at high blood levels, salicylates produce mechanistically and clinically distinct adverse effects that are fundamentally different from those occurring at lower therapeutic doses upon which its pharmacologic uses are based. These facts underscore that those studies conducted at doses exceeding a kinetic maximum—in this instance, first-order elimination process—are irrelevant and misleading for the purpose of understanding toxicity at lower therapeutic doses.

### Example #2: ethanol

Salicylates are not unique in this respect. The CNS-depressant effects of ethanol are also high-dose effects that occur secondary to saturation of metabolic capacity and the resultant change from first-order to zero-order kinetics (Høiseth et al. 2016; Jones 2010; Norberg et al. 2003). The CNS toxicity of ethanol, for which it is intentionally consumed as a social inebriant, depends upon sufficient concentrations in brain to perturb nerve cell membrane viscosity, slow neurotransmission, and inhibit the activity of GABAergic neurons and other receptor signaling pathways in the CNS (Kashem et al. 2021). At low consumption rates, ethanol does not reach CNS-depressant levels in brain due to first pass liver metabolism, which prevents its concentrations from accumulating in blood.

The rate-limiting step in ethyl alcohol metabolism is its conversion to acetaldehyde via the enzyme alcohol dehydrogenase (ADH), a liver enzyme with high affinity (a very low Km) but low capacity that becomes saturated with consumption of one or two standard alcoholic beverages per hour, or about 14–28 g ethanol per hour in an adult male (Høiseth et al. 2016; Jones 2010; Norberg et al. 2003). At consumption levels below this, the rate of ethanol metabolism is proportional to the blood level (i.e., elimination behaves as a first-order process) because sufficient ADH is present to quantitatively convert ethanol to acetaldehyde. Thus, at low consumptions levels, blood ethanol concentrations remain consistently very low. If ethanol consumption exceeds the available ADH, the capacity of this rate-limiting enzyme is saturated and ethanol metabolism becomes increasingly dependent upon CYP2E1, an inducible enzyme with higher capacity but lower affinity for alcohol (high Km). Under these conditions, ethanol metabolism as well as its disappearance from the blood becomes independent of the blood ethanol concentration. Elimination then behaves as a zero-order process equal to the maximum capacity of the enzymes that metabolize ethanol. Consequently, blood ethanol concentrations increase disproportionately, causing CNS concentrations to reach depressant levels (Høiseth et al. 2016; Jones 2010; Norberg et al. 2003). Without saturation of alcohol metabolism by ADH, rates of alcohol consumption typical in social settings would have little acute effect on people other than to increase urination frequency.

Most relevant to the point of this paper, if the hazard identification and risk problem formulation questions are intended to understand human health effects associated with chronic, high-dose human ethanol consumption, MTD animal toxicity testing would indeed be appropriate (although unjustified given the very large human cohort available for study of diseases associated with high-dose ethanol consumption). In contrast, if hazard identification and risk problem formulation is intended to address the very much lower ethanol exposures from occupational and other environmental scenarios, then chronic toxicity testing based on an MTD is clearly not relevant. In fact, MTD-based testing would provide misinformation because the hazards and risks associated with a sub-KMD-based dosing strategy consistent with realistic occupational and general environmental exposures are well-separated from intentional high-dose chronic drinking scenarios and their consequent kinetic differences. Importantly, the Heringa et al. (2020a), Slob et al. (2020) and Woutersen et al. (2020) series of papers would incorrectly imply that toxicity and hazard associated with very high-dose ethanol consumption informs hazard, toxicity and risk from much lower consumption levels; it certainly does not, even though MTD studies will inform toxicity and hazards of chronic ethanol abuse scenarios.

## KMD versus MTD

From these two examples, and many others that could be provided such as the example of chloroform-induced liver and kidney tumors discussed earlier in this review, it is clear that drug and chemical absorption, metabolism and elimination can be critical determinants of the type of toxicity exhibited, and that the nature of the toxicity exhibited by drugs and chemicals may differ drastically depending upon whether the dosage received is within the capacity of the organism to metabolize and eliminate the chemical, or exceeds it, i.e., is saturated. It, therefore, makes immanent sense that toxicology studies should be conducted with at least a rudimentary knowledge of the relationship between administered doses and the resultant blood levels. Instead of conducting studies at a so-called MTD, where overt toxic effects become evident, it would be more logical to conduct regulatory toxicology studies at doses up to those at which the organism’s processing of the chemical is altered, i.e., up to a kinetically determined maximum dose, or “KMD.”

Herein, the KMD is defined as the maximum external dose at which the toxicokinetics of a chemical remain unchanged relative to lower doses. Its estimation depends upon the ability to measure toxicokinetic changes in the test species under the same conditions used in toxicity studies, i.e., the internal dose, and the spacing of the external doses. Although it may seem obvious that except when realistic or foreseeable human exposures are reasonably close to the MTD, the KMD is superior to the MTD as a basis for dose selection in regulatory toxicity testing, it is necessary to provide some additional clarification regarding the phenomenon of kinetic alteration and saturation, as these concepts tend to be misunderstood and/or mischaracterized in discussions regarding the use of kinetics in dose-setting.

### Saturation is a threshold event, not a process

In pharmacology and toxicology, “saturation” refers to a state in which the concentration of chemical exceeds the concentration of metabolizing enzymes present in the system (Andersen 1981). At dosages that produce a saturated state, the rate at which chemicals are metabolized and/or eliminated will be altered compared to lower dosages. The parameter that is relevant to this alteration is the relationship between the administered dose and the blood level. “Saturation” does not refer to the proportion of the particular enzyme^2^ that is occupied as the dose of a substrate drug or chemical increases.

A simple analogy illustrates this concept. As a bathtub faucet is opened incrementally from a trickle to full flow, there is a corresponding process of continuous increase in the fractional capacity of the drain utilized to eliminate the water. However, there is no change in the water level in the tub unlesst the amount of water flowing into the tub exceeds the capacity of the drain to eliminate it. Like exceeding the capacity of a bathtub drain to eliminate water, saturation refers to the state in which dosage rate exceeds the capacity of the metabolic pathway to eliminate chemical, not to the continuous increase in the fractional capacity of the enzyme system that the body utilizes before the substrate concentration approaches 100% of the enzyme capacity, at which the system exhibits saturation behavior.

This concept is well described by the system of differential equations Renwick (1989) used to explain the implications of Michaelis–Menten (MM) enzyme kinetics for the onset of nonlinear TK (i.e., saturation), where C is the substrate concentration, Vm is the maximum rate of the enzymatic reaction, and Km is the affinity constant of the substrate for the enzyme:

1 $${\text{dC/dt = VmC/Km + C,}}$$

2 $${\text{dC/dt = VmC/Km,}}$$

3 $${\text{dC/dt = VmC/C = Vm}}{.}$$

Renwick explains that when substrate concentration is well below the Km (50% saturation of the enzyme), Eq. 1 reduces to Eq. 2, which is equivalent to the first-order kinetic rate constant, *k*_1_. When the substrate concentration greatly exceeds Km, Eq. 1 reduces to Eq. 3, which is the *V*max, a state at which total enzyme metabolism is limited to its maximum capacity, and zero-order kinetic behavior prevails.

#### Ethanol consumption illustrates why saturation is a threshold event

The toxicological significance of this difference is also illustrated by the example of ethanol metabolism and CNS toxicity in humans. It should be noted that this example is used only to illustrate kinetic principles and is not intended to equate social alcohol consumption with exposure to other chemicals, or to imply any recommendations about the safe consumption of alcoholic beverages for driving or any other purpose. The social use of ethanol intends to achieve inebriating (i.e., toxic) effects rather than to avoid them, but the kinetic principles apply regardless.

Ethanol elimination exhibits a zero-order kinetic profile at blood ethanol concentrations that produce overt CNS effects. Depending upon the CNS function or activity assessed, the minimum blood concentration of ethyl alcohol necessary to produce a measurable effect can be in the range of 0.022–0.05 g of ethanol per deciliter of blood, typically referred to as the “blood alcohol concentration” (BAC) in “grams percent” (g%) units. A BAC of 0.08 g% is considered presumptive evidence of intoxication for operation of an automobile in most U.S. states, and is lower in many European countries. It has been determined that a BAC of in the range of 0.017–0.022 g% saturates the enzymes that metabolize ethanol in humans (Høiseth et al. 2016; Jones 2010).

The analysis of Høiseth et al. (2016), shown in figure 2 of their publication, allowed us to extrapolate an ethanol elimination rate of 0.056 g%/h at a BAC of 0.08 g% under the assumption that saturation does not occur, and that the elimination rate continues to increase with increasing BAC according to an approximate first-order process. BACs were estimated for a 5-h drinking scenario under a first-order rate assumption. Those BACs were compared to BACs expected using an alcohol elimination rate near the high end of published elimination rates for non-alcoholics (Jones 2010; Norberg et al. 2003). The latter conforms to the zero-order kinetic elimination behavior by which ethanol is known to be eliminated in humans at BACs above about 0.02 g%, at which metabolic capacity is saturated (Table 1). The total body water method of Watson et al. (1981) was used to estimate BACs for a 40-year-old male of average size.

**Table 1:** Data for Fig. 1: 40-year-old male, 68 inches tall, 160 lbs

Drinking variables	Pre-consumption	Consumption 1	Consumption 2	Consumption 3	Consumption 4
% alcohol of drink	0.4	0.4	0.4	0.4	0.4
Fluid ounces per drink	1.5	1.5	1.5	1.5	1.5
Number of drinks	0	3	2	2	2
Time (hr) since last drink	0	1	1	1	1
Grams alcohol	0	42.04854	28.03236	28.03236	28.03236
BAC first-order elimination	0.000000	0.080881	0.078801	0.076721	0.074642
BAC high zero-order eliminiation	0.000000	0.080881	0.114801	0.148721	0.182642

Chemical and physiological parameters	
29.57	mls. per fluid ounce
0.79	Specific. Gravity Ethanol
72.576	Body weight in kg
172.72	Height in cm
0.01	Slow zero-order elimination rate (g%/h)
0.02	High zero-order elimination rate (g%/h)
0.056	First-order elimination rate (g%/h) at 0.08 g%

Calculated quantities	
Water content of blood (B)	0.8
TBW (Liters)	41.5907792
TBW (Deciliters)	415.907792
B/TBW Quotient	0.001923503

Blood alcohol concentrations (BAC) resulting from consumption of three standard alcoholic beverages (Consumption 1) followed by 2 alcoholic beverages every hour for 3 consecutive hours (Consumption 2, 3, 4) assuming either first-order or zero-order elimination kinetics

BACs were calculated by the Total Body Water (TBW) method of Watson et al. (1981) using the following formula:

Male Total Body Water (TBW) Volume [70.4% confidence interval (Watson et al. 1980)] = 2.447–0.09516 (age in yrs) + 0.1074 (height in cm) + 0.3362 (weight in kg).  Underlined values are independent (entered) variables; values not underlined are dependent (calculated) variables

A zero-order alcohol elimination rate of 0.2 g percent per hour was assumed, which represents a rate near the high end of the normal range for non-alcoholic adults (Jones 2010; Norberg et al. 2003). A first-order alcohol elimination rate of 0.056 g percent per hour was interpolated from the data found in Fig. 2 of the publication by Høiseth et al. (2016)

The alcohol content of a standard alcoholic beverage consisting of 1.5 oz of 80 proof (40%) ethanol was calculated as follows: (#drinks) (ounces per drink) (% alcohol) (29.57 ml per fl. oz.) (0.79 g alcohol per milliliter) = grams alcohol total

**Fig. 1:**
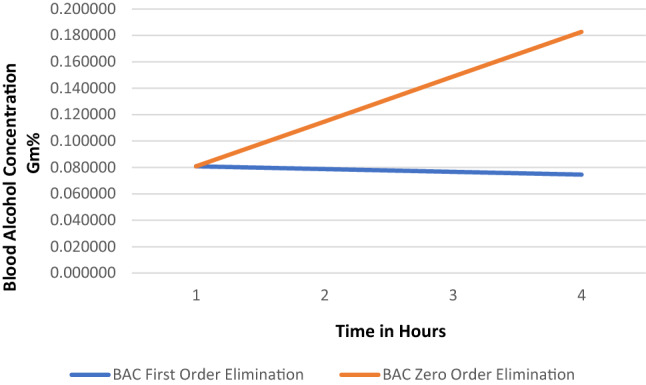
Non-saturation (first-order) versus saturation (zero-order) ethanol elimination kinetics. This figure shows blood alcohol concentrations (BACs) resulting from repeated ethanol consumption using theoretical non-saturation (first-order: blue line) versus actual saturation (zero-order: orange line) ethanol elimination kinetics for a hypothetical 40-year-old male, 68 inches tall, 160 lbs using data and equations shown in Table 1. Gm% = grams alcohol per deciliter of blood

Figure 1 provides BACs calculated for a hypothetical adult male following repeated ethanol consumption using theoretical non-saturation (first-order) versus actual saturation (zero-order) ethanol elimination kinetics. Figure 1 shows that if saturation of metabolism were a process rather than a threshold condition, after achieving an initial BAC of about 0.08 g%, as would be expected after rapid consumption of about three standard alcoholic drinks (Consumption 1), the subject’s BAC would decline below the 0.08 g% presumptive legal driving limit despite continuing to drink 2 alcoholic beverages per hour (Consumptions 2–4). On the contrary, it is well established that even if that individual were a rapid metabolizer of ethanol, eliminating 0.02 g%/h by zero-order kinetics (normal range = 0.01–0.02 g%/h), his BAC would rise continuously with successive consumption of 2 drinks per hour, producing an excessive level of intoxication well beyond the initial BAC of 0.08 g% (Consumption 1) within a few hours. This quantitative example demonstrates that, although the continual increase in fractional enzyme capacity utilized with increasing chemical concentration is indeed a process that begins with administration of even the low doses, this process is irrelevant to whether saturation is an observable event, and thus, whether the KMD is a useful concept for dose-setting in toxicology testing.

### Inflection points are irrelevant

In asserting that saturation is a continuous process rather than a threshold condition, much argumentation has been made based on the presumption that a threshold event would produce an unambiguous inflection point in the administered-dose/blood-concentration relationship (Heringa et al. 2020a, b, c; Slob et al. 2020; Woutersen et al. 2020). Although the empirical basis of Heringa et al.’s claim that “*a sharp inflection point is not observable in most instances*” has been challenged (Sewell et al. 2020; Smith and Perfetti 2020; Terry et al. 2020), a challenge to which the authors partially responded (Heringa et al. 2020b, c), our focus is on their conclusion that imprecision in the location of an inflection point means that saturation of metabolism must be a non-threshold, continuous process. Several factors may contribute to uncertainty in the precise location of an inflection point, including primarily the number of doses used to estimate the kinetic relationship and the spacing of those doses, and—unless enough animals are evaluated to ensure statistical power—biological variability. This uncertainty should not obscure the fact that biological systems often, but not always, respond distinctly differently to high versus low doses of a chemical or physical agent, with no indication of high-dose effects occurring below a threshold dose. Indeed, the field of pharmacology has successfully dealt with the issue of uncertainty in inflection points without resorting to assumptions that cannot be validated, such as the assumption that the inability to observe a precise inflection point precludes a threshold.

The uncertainty of the determination depends on the dose-spacing employed in the study relative to the dose at which kinetic changes occur, not upon the validity of established knowledge that toxicity is kinetically dependent. Returning to our bathtub analogy, assume that the capacity of the drain is 1 gallon per minute (gal/min), but is as yet unknown to the experimenter. Assume that inputs of 0.4 and 0.8 gal/min are observed by experiment to be linearly related, i.e., no accumulation of water in the tub, and that an input of 1.6 gal/min produces accumulation of water in the tub. These data would leave considerable uncertainty as to whether 1 gal/min or 1.5 gal/min is the better estimate of drain capacity. If, however, the third input had shown that 1.2 gal/min produced accumulation of water in the tub, the data would yield an estimate of drain capacity closer to the true value of 1 gal/min. Nonetheless, both data sets provide high confidence that an input of 1.6 gal/min exceeds the drain capacity as it would be impossible for water to accumulate in the tub had saturation not occurred at both 1.2 and 1.6 gal/min.

#### Example: Slob et al. (2020), Fig. 8

**Fig. 2:**
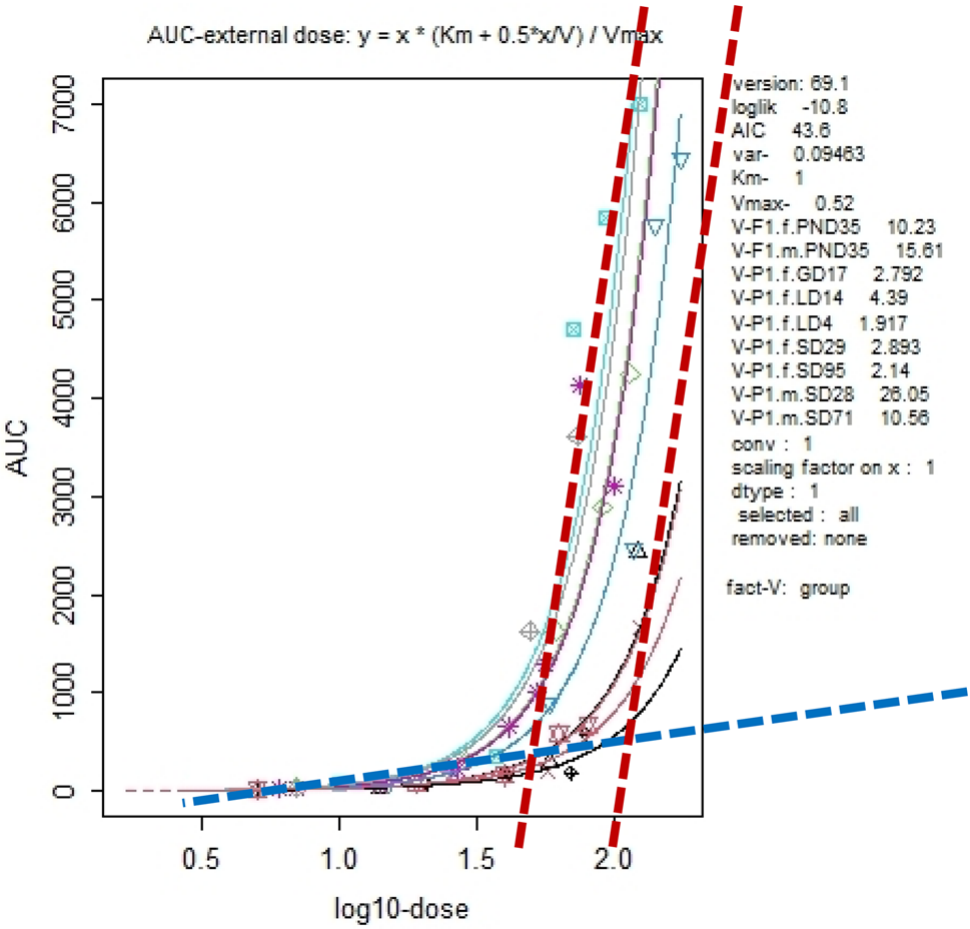
KMD Region Identified in AUC-External dose plot from Figure 8(a) of Slob et al. 2020. Figure 8 of Slob et al. 2020 showing the relationship between area under the blood concentration curve (AUC) for 2,4-D plotted against the base 10 logarithm of the dose administered to rats. The blue dashed line is an estimate of the slope of the relationship at doses below a log10-dose of approximately 1.6, across which the slope appears to be stable. Red dashed lines are estimates of the slope of the relationship in the dose range of log10-dose 1.6–2.0

To clarify our argument that uncertainty in the location of an inflection point should not preclude identifying changes in toxicokinetic behavior at high versus low doses, we consider Slob et al.’s (2020) reanalysis of a select subset of data from Saghir et al. (2013) in which they plotted the AUC of blood concentrations versus increasing administered doses of 2,4D (Fig. 8 in Slob et al. 2020). Because their plots did not exhibit sharp inflection points, Slob et al. (2020) interpreted this as showing the continuous nature of the change in the relationship between administered-dose to blood-level and thus, the lack of a KMD. In Fig. 2, we show that although Slob et al.’s plots lack a sharp inflection point, there is nonetheless a clear difference in the relationship between the blood level and high versus low administered doses, as evidenced by a prominent change in slope that occurs between log dose 1.6 and 2.0. Hence, imprecision in the location of an inflection point does not obscure the fact that blood concentrations bear a different relationship to low versus high administered doses, which is a clear indication that the biological system handles low doses of 2,4D fundamentally differently than high doses.

Because experimental data provide only a vague representation of the underlying biological system rather than an exact replication, it is unreasonable to require a mathematically distinct inflection point to infer a change in the biological response. Rather than a distinct point of inflection or an abrupt increase or decrease in the response parameter, the dose range at which biological changes occur can be identified through the point of maximum curvature, a method explained in greater detail in a companion to this paper (Burgoon et al. 2021; in preparation). This region of maximum curvature can be defined as the KMD region.

### Changes in the relationship between administered-dose and blood concentration are critical

For purposes of toxicological interpretation and regulatory toxicology study designs, it is critical to understand how the region of the administered dose / blood concentration curve at which toxicological effects are measured relates to the kinetics of the chemical, and whether this region is below, above, or within the area of maximum curvature, i.e., the KMD region. It is not to be inferred, however, that adverse effects cannot occur below a KMD, or that adverse effects will always manifest just above a KMD; the point is that toxicity and its underlying mechanisms can be expected to differ with a change in kinetics. Effects produced at doses either above or below the KMD region are most clearly interpretable and relevant for the purpose of defining the non-hazardous dose range. Results produced at doses within the KMD range or across doses that span it are likely ambiguous for the purpose of establishing safety as well as for making inferences about the potential modes of action underlying toxic effects. On the other hand, toxicity observed at doses clearly above the KMD region can be unambiguously interpreted if the KMD region is well separated from the range of foreseeable human exposures. Such toxicity lacks quantitative dosimetric and mechanistic relevance to humans and requires no further experimental attention as it represents an adverse effect confounded by overloading of the animal’s physiological and metabolic capacity. In this regard, the KMD may be superior to more nuanced signs of overstress, such as body weight or histopathological alterations typically used to identify an MTD, since kinetic alterations are a clear indication that an animal’s capacity for metabolism and/or clearance of the chemical has been exceeded (Bus 2017).

A final point that should not be lost or mischaracterized: not every chemical exhibits a point of saturation, a change in slope, or a KMD in its administered-dose/blood-concentration relationship. For new regulatory toxicity testing, it is critical to know which chemicals do, and which do not exhibit those kinetic characteristics and to incorporate this understanding into toxicological study designs. For existing toxicology data that were generated without utilizing kinetic understanding in the study design, interpretations about relevant hazards and adverse effects would be informed, and potentially corrected, by kinetic data. Failing to perform kinetic studies and the understanding that can be gleaned from them will ensure that regulatory toxicology studies continue to maximize uncertainty, inefficiency, waste of animal lives, and animal suffering.

## Conclusions

A primary goal of toxicology in the twenty-first century should be to maximize use of kinetic understanding to meet the goals of regulatory toxicology, which are to define the range of exposures and doses at which chemicals can be used safely. In vivo KMD data are necessary for interpreting the risk relevance of responses observed from in vitro and high-throughput studies, which carry limited, if any, relevance for human risk when the concentrations at which responses are observed exceed the blood/tissue concentrations produced at the KMD as identified by in vivo TK studies. Focusing on so-called “intrinsic hazards” rather than on safe doses ranges is illogical because hazards are not “intrinsic.” All chemicals—natural or synthetic, endogenous or exogenous—exhibit toxicity (hazard), the manifestation of which is always dose-dependent (McCarty et al. 2020). Unless it is imagined that some chemicals lack hazards, such a focus wastes time and effort because it simply confirms what is already known. To paraphrase Paracelsus, ‘there are no safe chemicals; there are only safe exposures and doses.’ Therefore, identifying and characterizing safe exposures and doses should be the goal of regulatory toxicology. The argument that kinetics is not an adverse effect and so should not provide the basis for dose selection seems equally irrational. Instead, because a kinetic change is not an adverse effect per se but precedes adverse effects by driving the systemic dose and thereby determining toxicity, kinetic changes would seem to provide a much better basis for protection of health than the observation of overt adverse effects.

Not only are the effects of chemicals dose-dependent, but the mechanisms of action that produce those effects are also dose-dependent. Kinetics often underlies the dose-dependency of both mechanisms and effects. Therefore, an understanding of kinetics and its use in dose-setting for regulatory toxicity testing is biologically sound, theoretically logical, and appropriate. Not only will dose-setting based on kinetic understanding improve the human relevance of toxicity testing results, but it will also increase the efficiency of toxicity testing, clarify the interpretation of results and reduce unnecessary animal use and suffering.

Arguments against the use of PK/TK in dose-setting often derive from an overly restrictive interpretation of the relationship between PK/TK and biological effects. Uncertainty regarding points of inflection in the relationship between administered dose and blood concentration of a chemical does not logically translate to a lack of saturation, to saturation being a continuous process, or to a lack of saturation above a certain chemical concentration, i.e., a threshold. As noted previously, when the chemical concentration greatly exceeds the *Km* of metabolizing enzymes, the rate of biotransformation approximates the *V*max and biotransformation reverts to zero-order kinetics. Rather than precision with respect to an inflection point, the salient issue is whether there is a biologically significant change in the relationship between administered dose and blood concentration at low versus high doses. For many chemicals, but not all, such differences exist and underly the dose-dependency of mechanisms and effects. An understanding of PK/TK is critical to identifying those chemicals that do, and those that do not, exhibit such dose dependencies. It is indisputable that kinetic changes drive changes in systemic dose, which in turn are fundamental determinates of whether and how toxicity occurs. The coupling of expanded TK information with that of advancing human exposure science offers substantial opportunities for improving the human relevance of toxicity testing protocols.

For many chemicals, but not all, a finite range of administered doses can be identified that separates a biologically significant difference in the relationship between administered dose and blood concentration changes. Within this range lies the Kinetic Maximum Dose, or KMD, defined as the maximum external dose at which the toxicokinetics of a chemical remain unchanged relative to lower doses. An alternative method for identifying the KMD based on changes in slope and maximum curvature of the administered dose/blood concentration relations is the topic of a companion paper (Burgoon et al. 2021). This method obviates recent criticisms of the KMD approach (Heringa et al. 2020a, b, c; Slob et al. 2020; Woutersen et al. 2020) and offers advantages that will increase confidence regarding the safe dose range and reduce unnecessary use of animals in regulatory toxicity testing.

The pharmacological and toxicological advancements made possible by PK/TK have been formidable, as described herein. Although general acceptance of those advancements has required considerable time, there is no longer controversy regarding their contribution to pharmacological and toxicological understanding and their value to the applied technologies that rely upon them. The relationship between toxicity and aspects of TK, such as saturable metabolism, was described 40 years ago (Andersen 1981), and those relationships have been verified in numerous ways over the ensuing decades. Thus, it should no longer be controversial that PK/TK offers a biologically valid means of improving the way doses are selected for regulatory toxicology studies. The time has come for regulatory toxicology to embrace the improved biological understanding made possible by proper application of PK/TK. Continued resistance will only help to ensure that regulatory toxicology remains an observational science dependent upon default assumptions rather than biological knowledge to project hazard across species and orders-of-magnitude differences in dose.

## Abbreviations

AUC: Area under the curve
BAC: Blood alcohol concentration
KMD: Kinetic maximum dose
MoA: Mode of action
MTD: Maximum tolerated dose
PK: Pharmacokinetics
TK: Toxicokinetics

## Footnotes

1 The name aspirin is used for brevity, understanding that the pharmacological and toxicological effects of acetyl salicylate are due in part to its active metabolite salicylic acid and other salicylates.

2 For simplicity, drug-metabolizing enzymes are used as examples, but the same concepts apply to saturation of receptors, transporters, etc.
